# Factors influencing research productivity among Syrian medical professionals amidst conflict: a case-control study

**DOI:** 10.1186/s12909-024-05681-y

**Published:** 2024-07-11

**Authors:** Ibrahem Hanafi, Kheder Kheder, Rami Sabouni, Ahmad Rami Rahmeh, Marah Alsalkini, Mouaz Hanafi, Ahmad Naeem, Fares Alahdab

**Affiliations:** 1 Division of Neurology, Department of Internal Medicine, Faculty of Medicine, Damascus University, Mazzah, Damascus, Syria; 2 Faculty of Medicine, Al Andalus University, Tartus, Syria; 3 Faculty of Medicine, Damascus University, Damascus, Syria; 4 Department of Ophthalmology, Aleppo University Hospital, Aleppo, Syria; 5 Faculty of Medicine, Albaath University, Homs, Syria; 6 Department of Oral and Maxillofacial Surgery, Faculty of Dentistry, Damascus University, Damascus, Syria; 7 Houston Methodist Academic Institute, Houston, TX USA; 8 Weill Cornell Medical College, New York, NY USA; 9 School of Biomedical Informatics, University of Texas, Houston, TX USA

**Keywords:** Research productivity, Publications, Research attitudes, Research barriers, Research-related knowledge, Syrian authors, Syrian crisis

## Abstract

**Background:**

Medical research productivity is globally increasing, with a lagging progress in third-world countries due to significant challenges, including inadequate training and brain drain. Syria had been showing a slow upward trend until the war broke out and severely hindered academic growth and productivity. A deeper understanding of the factors influencing research productivity in this context are fundamental to guide educational policies and resource allocation. Previous cross-sectional studies that evaluated the perspectives of Syrian academics on the issue were limited by the small sample size of published healthcare workers, making it difficult to identify the factors that enabled them to pursue research.

**Methods:**

To address this challenge, we employed a case-control design. We isolated published early-career Syrian healthcare workers and compared their characteristics and perceptions to unpublished matched controls. Authors in the fields of medicine, dentistry, and pharmacy affiliated with any Syrian University were identified through an extensive search of PubMed and Google Scholar.These authors were invited to complete a questionnaire that covered participants’ research contributions, alongside their self-assessed knowledge, attitudes, and barriers towards research. The questionnaire was publicly published to recruit an equal sample of matching controls, with half consisting of unpublished researchers and the other half of participants without prior research contributions.

**Results:**

Six-hundred-sixteen participants were recruited. Their knowledge, attitudes, and perceived barriers explained 46% and 34% of the variability in research involvement and publication, respectively (*P* < 0.001). Getting involved in and publishing research studies associated with higher research-related knowledge and attitudes (*P* < 0.001). Respondents’ assessment of research-related barriers and their academic scores did not differ between cases and controls. Superior research-related knowledge and attitudes were associated with male gender, higher English competency, and better internet connectivity. Meanwhile, extracurricular training and mentors’ support were associated with more positive research-related attitudes and less perceived barriers.

**Conclusions:**

Research productivity of medical professionals in Syria exhibits a positive correlation with their knowledge and favorable attitudes towards medical research. Noteworthy, the demographic variations are linked to disparities in research-related knowledge and motivation. In conclusion, these results suggest a potential avenue for enhancement through concentrated efforts on improving extracurricular training interventions and mentors’ support.

**Supplementary Information:**

The online version contains supplementary material available at 10.1186/s12909-024-05681-y.

## Background

Research productivity in lower- and middle-income countries (LMICs), especially in the Middle East and North Africa, is hindered by many fundamental and economic challenges (i.e., lack of funding and research facilities) [1–3]. Furthermore, LMIC researchers face language barriers that often make it difficult to publish research in higher-tier academic journals as well as collaborate internationally [1, 3]. Protracted conflicts and the lack of resources also exacerbate the drain of professional medical academic personnel from the region [1], increasing the responsibilities of physicians under training who remain [4–7]. In addition to the lack of sufficient training and mentorship, residents and medical students face time shortages due to burdensome educational tasks and overwhelming working hours, which is further intensified by the aforementioned drain of trained medical personnel [8, 9].

The level of medical research in Syria has been modest, when compared to neighboring countries in the region, with a slow upward trend over the past several decades [10, 11]. On top of that, the long-term continuing armed conflict destroyed and depleted much of the medical and educational facilities around the country [12, 13]. The dreadful life circumstances and political insecurity also led a massive proportion of healthcare professionals to flee the country [14]. This shortage of staff forced the remaining physicians and centers to shoulder the burden of the immense flow of patients [15], depleting their capacity to pursue research. Although more awareness and higher attitudes towards medical research were reported recently in Syria [16–18], most of the research duties were handled by independent residents and medical students, without sufficient institutional support and mentorship [19]. Unsurprisingly, they mostly published case reports and simple cross-sectional studies as they tried their best to work with what they had available [16–18].

The primary step towards enhancing research development in Syria is the identification and empowerment of the factors that enabled authors from Syria to engage in research despite the challenging environment. To begin with, an in-depth investigation of the knowledge, attitudes, practices, and perceived barriers is fundamental as a commonly used method to unearth the root causes of limited research productivity [8, 16, 18, 20]. Furthermore, well-documented evidence highlighted the impact of demographic factors such as gender [21, 22], university [8, 20], specialty [23], and English proficiency [24] on research productivity as well as knowledge, attitudes, and barriers related to research. Thus, a comprehensive multi-dimensional assessment is required to strategize interventions that may improve the current academic research realities.

Previous cross-sectional investigations examining attitudes and perceived barriers towards research among Syrian medical under- and postgraduate students have typically involved a limited number of students with prior publications [16, 18]. In our case-control study, we targeted the entire cohort of published authors in Syria and compared them with matching unpublished controls. Our inquiry encompassed an exploration of research-related attitudes, knowledge, perceived barriers in both study groups, as well as an analysis of the demographic characteristics associated with these factors.

## Methods

### Study design and population

This questionnaire-based case-control study targeted medical personnel between the 3rd year of undergraduate training and freshly graduated specialists in all healthcare-related institutions and universities around Syria. Briefly, five schools, which were the largest in the country, dominated the contributions to medical research in Syria. Three of these (Damascus, Aleppo, and Tishreen [Latakia]) are the only ones with graduate programs. The other medical schools were not excluded; however, they were either recently established with very small classes or private universities without affiliated medical centers. Consequently, most graduates from these smaller institutions continued their education at the larger universities. This situation also contributed to a lower interest in research activities in the smaller institutions [16]. Nonetheless, we included a few participants from smaller universities who conducted their research at the medical centers affiliated with the larger universities. They were therefore grouped together with the students of the university where they performed their research.

No sample size calculation was performed for this study because of the limited number of published authors, who were all targeted. First, we identified the cases (i.e., published authors) by a systematic search of PubMed and Google Scholar for all authors in the fields of medicine, dentistry, and pharmacy with affiliation to any Syrian university. We enrolled only early-career authors of the identified list excluding specialists who concluded their postgraduate studies more than one year before the time of data collection. The exclusion was performed by personal communication with each one of the identified cases individually. After collecting the responses from the cases, we enrolled an equal sample of unpublished controls. The decision to match cases to controls on a 1:1 ratio was made to ensure a straightforward, balanced, and statistically powerful comparison between the arms. The matching between the cases and the controls was stratified based on the university, field (i.e., medicine, dentistry, or pharmacy), and educational level (i.e., undergraduate or postgraduate studies). For a more detailed analysis, the cases were divided into two equal groups: (i) those with more than one publication and (ii) those with only one publication. Similarly, the controls were recruited to form two equal groups: (iii) those involved in at least one research study but without any publications and (iv) those not involved in any research activities.

The electronic questionnaire was individually communicated to published authors (i.e., the first two arms). A reminder was sent to these authors two weeks after the first communication of the questionnaire in case they did not confirm their participation. Control participants (i.e., the third and fourth arms) were collected after public dissemination of the questionnaire on social media groups of students and junior personnel. These groups are the most common form of communication among students, serving as a platform for sharing both curricular and extracurricular scientific information.The data were collected between April and May 2021 using an online English questionnaire created using Google Forms. Participation was voluntary and the participants were briefed about the aims of the study. Once informed consent for participation was obtained (which was on the first page of the questionnaire), a participant was included in the study. This study was approved by the institutional review board of Damascus University, and it complied with the declaration of Helsinki as revised in 2013 [25].

### The questionnaire

The questionnaire used in this study consisted of two sections: The first section investigated demographic determinants including gender, university, specialty, self-assessed English competence, undergraduate averaged academic score, and the number of research projects involved in or published. The second portion of the questionnaire included self-assessment of the participants’ knowledge of, attitudes towards, and perceived barriers around getting involved in academic research. The attitudes and barriers items of the second section were reused from our previous published studies [16, 18], with amendments based on their findings.

Knowledge assessment consisted of nine subjective questions covering participants’ competence in understanding and evaluating scientific research publications as well as planning, conducting, and publishing scientific articles using a 5-point Likert scale ranging from poor (1) to advanced (5) knowledge.

The final version of the questionnaire was piloted on a sample of twenty participants from different specialties and academic levels of the targeted population to confirm that they could follow the instructions without difficulties in the language, comprehension, or structure.

### Data analysis

The data were exported from Google Forms to Microsoft Excel and then imported into the Statistical Package for the Social Sciences (SPSS) software (version 23.0, SPSS Inc., Chicago, IL, United States). Al-Baath and Tartus Universities offer limited postgraduate healthcare-related programs, and the vast majority of their graduates join postgraduate studies in Damascus and Tishreen Universities, respectively. Therefore, and due to the few numbers of undergraduate participants in these universities, they were grouped with Damascus and Tishreen Universities’ pool, respectively. Categorical variables were presented as frequencies and percentages. Chi-square test was performed to assess the association between the demographic characteristics and participation-publication groups. Continuous variables were summarized as means and standard deviations. Independent samples Kruskal-Wallis non-parametric test was applied to compare the arms of the study regarding their total scores in knowledge, attitudes and barriers as well as their academic scores. It was also supplemented by post-hoc pairwise comparisons, with Bonferroni correction for multiple comparisons. Additionally, Spearman’s rho coefficients were used to test the correlations between continuous variables. Ordinal logistic regression was employed to analyze models of independent variables, encompassing participants’ knowledge, attitudes, and barriers to estimate their association with engagement in and publishing of research studies. The findings were conveyed through regression coefficients and their corresponding 95% confidence intervals (CI). An Alpha value of 0.05 was determined as a threshold of statistical significance with the use of Bonferroni correction for multiple comparisons.

Finally, the supplementary materials of this study included a further analysis applied on the data of a previous cross-sectional study that was conducted to investigate research attitudes and barriers among Syrian postgraduate students in different higher education universities of healthcare professions in July 2020 [16]. This further analysis sought to illustrate the connection between mentors’ support and extracurricular self-paced training regarding medical research on one hand and the attitudes and perceived barriers towards research involvement on the other [16].

## Results

A total number of 616 participants were recruited for the study; almost half of them were females (*n* = 330, 53.6%), and 80% affiliated with Damascus or Aleppo universities (*n* = 493). Their academic scores averaged 81.6 ± 6.06 (Table 1). Published authors who met the inclusion criteria were 407; 308 of them completed the study (cases’ response rate of 75.7%). Cases and controls were distributed homogeneously among universities, genders, and specialties. Getting involved in and publishing research was associated with at least upper-intermediate English writing skills (OR: 2.01 [1.39–2.90], and 2.94 [2.11–4.11], respectively; *P* < 0.001; Table 2).

**Table 1:** Composition and demographic characteristics of the sample

Variables	*N* (%)	Variables	*N* (%)
University		Specialty	
Damascus	247 (40.1)	Medicine undergraduate	284 (46.1)
Tishreen	123 (20)	Pharmacy undergraduate	10 (1.6)
Aleppo	246 (39.9)	Dentistry undergraduate	7 (1.1)
Gender		Internal medicine specialties^¶^	120 (19.5)
Female	330 (53.6)	Surgical specialties^†^	61 (9.9)
Male	286 (46.4)	Clinics specialties^‡^	59 (9.6)
Type of studies		Translational specialties^ǁ^	37 (6)
Undergraduate	301 (48.9)	Pharmacy postgraduate	19 (3.1)
Postgraduate at the Ministry of Higher Education	245 (39.8)	Dentistry postgraduate	19 (3.1)
Postgraduate at the Ministry of Health	49 (8)	Internet connection	
Postgraduate at the Ministry of Defense	6 (1)	No/bad internet connection	123 (20)
Postgraduate Abroad	15 (2.4)	Good internet connection	493 (80)
Academic level		Personal computer	
Undergraduate third year	15 (2.4)	No (share someone’s device)	85 (13.8)
Undergraduate fourth year	57 (9.3)	Yes	531 (86.2)
Undergraduate fifth year	82 (13.3)	English reading skills	
Undergraduate sixth year	105 (17)	A2 (Elementary)	24 (3.9)
Fresh graduate*	42 (6.8)	B1 (Intermediate)	145 (23.5)
Postgraduate first year	98 (15.9)	B2 (Upper Intermediate)	236 (38.3)
Postgraduate second year	68 (11)	C1 (Advanced)	211 (34.3)
Postgraduate third year	53 (8.6)	English writing skills	
Postgraduate fourth year	45 (7.3)	A2 (Elementary)	50 (8.2)
Postgraduate fifth year	19 (3.1)	B1 (Intermediate)	230 (37.3)
Fresh specialists*	32 (5.2)	B2 (Upper Intermediate)	224 (36.4)
		C1 (Advanced)	112 (18.2)

n: Number of participants who chose the corresponding answer, %: Percentage of participants who chose the corresponding answer; ¶ Including pediatrics and psychiatry; † Including obstetrics and gynecology, anesthesiology, and emergency medicine; ‡ Including ophthalmology, otolaryngology, and dermatology; II Including laboratory medicine, radiology, and pathology; * Fresh graduates refers to graduates who did not join a residency program yet, while fresh specialists are those who concluded their postgraduate studies no more than one year before data collection

**Table 2:** Associations between participants’ characteristics and conducting and publishing of research projects

Factor	Participation			Publication		
	n (%)	*P* Value^*^	OR [95%CI]^#^	n (%)	*P* Value^*^	OR [95%CI]^#^
University		0.041			0.141	
Aleppo	181 (73.6)			113 (45.9)		
Tishreen	83 (67.5)		0.75 [0.47–1.19]	44 (35.8)		0.67 [0.42–1.02]
Damascus	196 (79.4)		1.38 [0.91–2.10]	112 (45.3)		0.98 [0.69–1.39]
Gender		0.034			0.187	
Male	225 (78.7)		1.49 [1.03–2.16]	133 (46.5)		1.24 [0.90–1.71]
Female	235 (71.2)			136 (41.2)		
Academic level		0.001^§^			< 0.001^§^	
Postgraduate	254 (80.6)		1.92 [1.33–2.78]	168 (53.3)		2.12 [1.52–2.95]
Undergraduate	206 (68.4)			101 (33.6)		
Specialty		0.748			0.608	
Human medicine	420 (74.9)			248 (44.2)		
Pharmacy	20 (69.0)		0.75 [0.33–1.68]	12 (41.4)		0.89 [0.42–1.90]
Dentistry	20 (76.9)		1.12 [0.44–2.84]	9 (34.6)		0.67 [0.29–1.53]
Internet connection		0.372			0.077	
Good connection	372 (75.5)		1.22 [0.79–1.90]	224 (45.4)		1.44 [0.96–2.17]
No/bad connection	88 (71.5)			45 (36.6)		
Personal computer		0.005^§^			0.228	
No (shared device)	53 (62.4)			32 (37.6)		
Yes	407 (76.6)		1.98 [1.22–3.21]	237 (44.6)		1.34 [0.83–2.14]
English reading skills		0.020			< 0.001^§^	
≤ B1 (Intermediate)	115 (68.0)			51 (30.2)		
> B1 (Intermediate)	345 (77.2)		1.59 [1.07–2.35]	218 (48.8)		2.20 [1.51–3.21]
English writing skills		< 0.001^§^			< 0.001^§^	
≤ B1 (Intermediate)	189 (67.5)			83 (29.6)		
> B1 (Intermediate)	271 (80.7)		2.01 [1.39–2.90]	186 (55.4)		2.94 [2.11–4.11]

* Chi-square test; § Statistically significant P-value after Bonferroni correction for multiple comparisons (0.050/8 = 0.006) # Odds ratios (OR) and 95% confidence intervals (CI)

**Table 3:** Ordinal logistic regression predicts research productivity based on participants’ research-related knowledge, attitudes, and barriers

Model/item	Participation*	Publication*^#^
Knowledge, attitudes, and barriers	R^2^ = 0.46 (*P* < 0.001)†	R^2^ = 0.34 (*P* < 0.001)†
Knowledge	R^2^ = 0.41 (*P* < 0.001)†	R^2^ = 0.29 (*P* < 0.001)†
Comprehending scientific publications	-0.09 [-0.33,0.16]	-0.18 [-0.49,0.12]
Critical appraisal of scientific publications	-0.11 [-0.36,0.13]	-0.12 [-0.42,0.18]
Searching the medical literature	0.44 [0.21,0.67]^§^	0.16 [-0.14,0.45]
Identifying topics with lack of evidence	-0.03 [-0.26,0.19]	0.31 [0.04,0.59]^§^
Research methodology	-0.15 [-0.38,0.09]	-0.10 [-0.38,0.17]
Planning and conducting research	0.49 [0.25,0.73]^§^	0.15 [-0.14,0.44]
Statistical analysis	-0.29 [-0.48,-0.10]^§^	-0.35 [-0.58,-0.12]^§^
Academic writing	0.26 [0.04,0.48]^§^	0.12 [-0.15,0.39]
Publication process	0.69 [0.50,0.89]^§^	0.79 [0.56,1.02]^§^
Attitudes	R^2^ = 0.06 (*P* < 0.001)†	R^2^ = 0.03 (*P* = 0.054)†
Research is important for optimal patient care	0.06 [-0.22,0.33]	-0.00 [-0.34,0.33]
Research is important in the medical field	-0.02 [-0.31,0.26]	-0.19 [-0.54,0.17]
Research will be a part of my career goals	0.22 [0.02,0.43]^§^	0.10 [-0.15,0.36]
Research is not always costly	0.26 [0.12,0.40]^§^	0.21 [0.03,0.38]^§^
Teaching research methodology should be part of the curriculum	0.03 [-0.20,0.25]	-0.07 [-0.34,0.20]
Publishing scientific papers is important during medical studies	0.00 [-0.22,0.22]	0.21 [-0.06,0.47]
Barriers	R^2^ = 0.14 (*P* < 0.001)†	R^2^ = 0.06 (*P* = 0.018)†
Poor research attitudes of doctors	0.10 [-0.07,0.27]	0.15 [-0.05,0.35]
Poor research attitudes of patients	-0.11 [-0.27,0.04]	-0.11 [-0.28,0.07]
Lack of time	0.14 [-0.00,0.28]	0.15 [-0.02,0.32]
Lack of opportunities	-0.17 [-0.33,-0.01]^§^	-0.30 [-0.49,-0.12]^§^
Lack of training	-0.36 [-0.56,-0.15]^§^	0.04 [-0.20,0.28]
Lack of reward/motivation	0.33 [0.14,0.51]^§^	-0.03 [-0.25,0.19]
Lack of funding support	-0.09 [-0.28,0.11]	-0.04 [-0.26,0.19]
Lack of communication between students of the same specialties in different centers	-0.03 [-0.26,0.21]	0.05 [-0.23,0.33]
Lack of communication between students from different specialties	0.01 [-0.22,0.25]	0.05 [-0.22,0.33]
Poor documentation in patients’ records	0.59 [0.40,0.77]^§^	0.19 [-0.04,0.41]
Research mentors are not easily available	0.14 [-0.06,0.33]	0.02 [-0.21,0.25]
Difficult approvals to conduct research	-0.16 [-0.32,0.01]	0.10 [-0.09,0.29]
Language or internet limitations	-0.18 [-0.32,-0.05]^§^	-0.18 [-0.34,-0.02]^§^

* The numbers represent the regression coefficients calculated using ordinal logistic regression; # Excluding participants who never participated to any research project before (*n* = 460); † R^2^ of the Model fitting was calculate using the Nagelkerke methods; § significant independent predictor

**Fig. 1:**
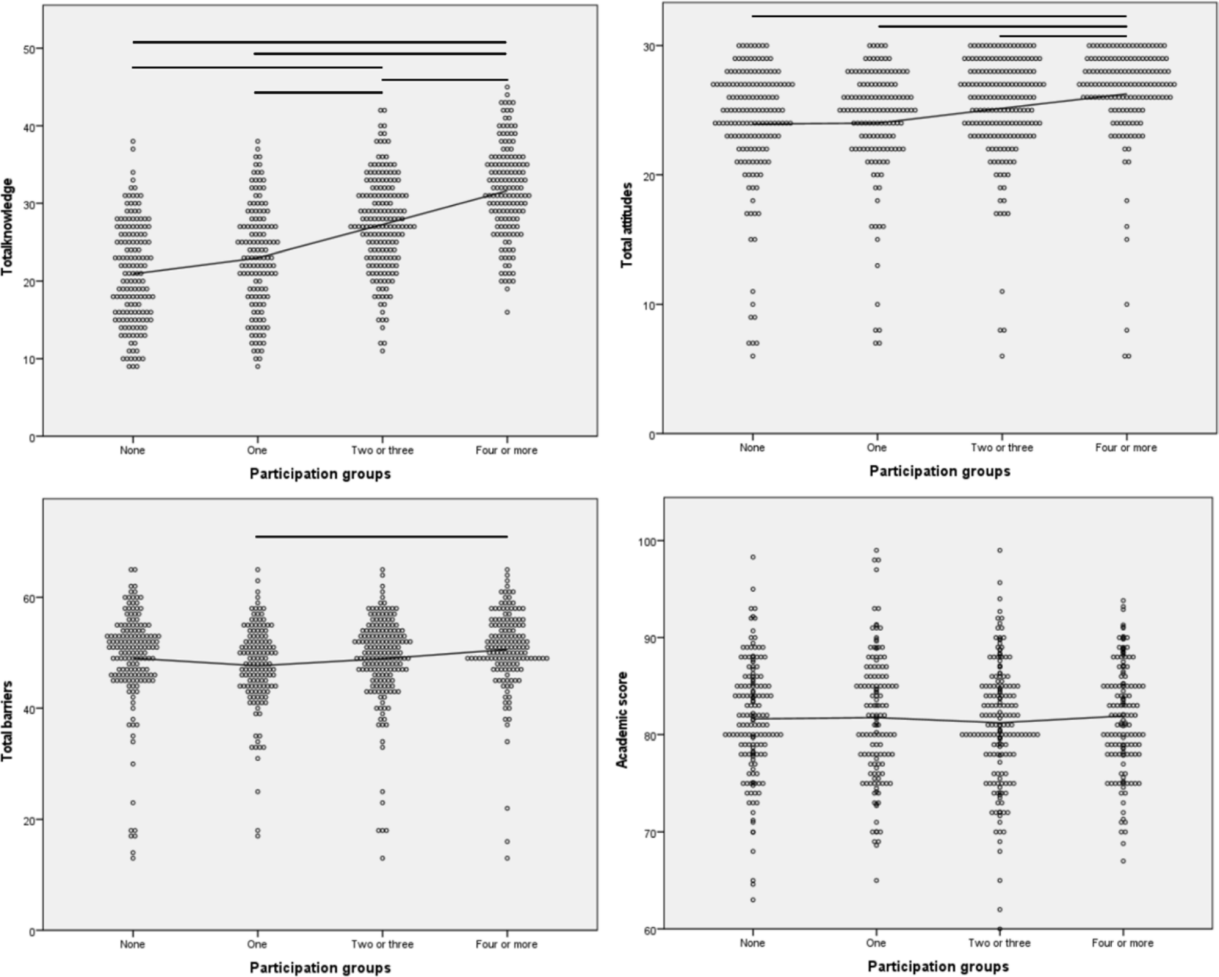
Association between the number of research projects and research-related knowledge, attitudes, barriers, and academic scores. Legend: Scatter point plot of participants total knowledge, attitudes, barriers, and academic scores against their level of participation in research studies. Independent samples Kruskal-Wallis non-parametric tests were conducted for each graph. An interpolation straight line was plotted for each graph as well. The horizontal lines illustrate only the statistically significant pairwise comparisons when the groups test was statistically significant. The significance level of pairwise comparisons were adjusted for multiple comparisons

**Fig. 2:**
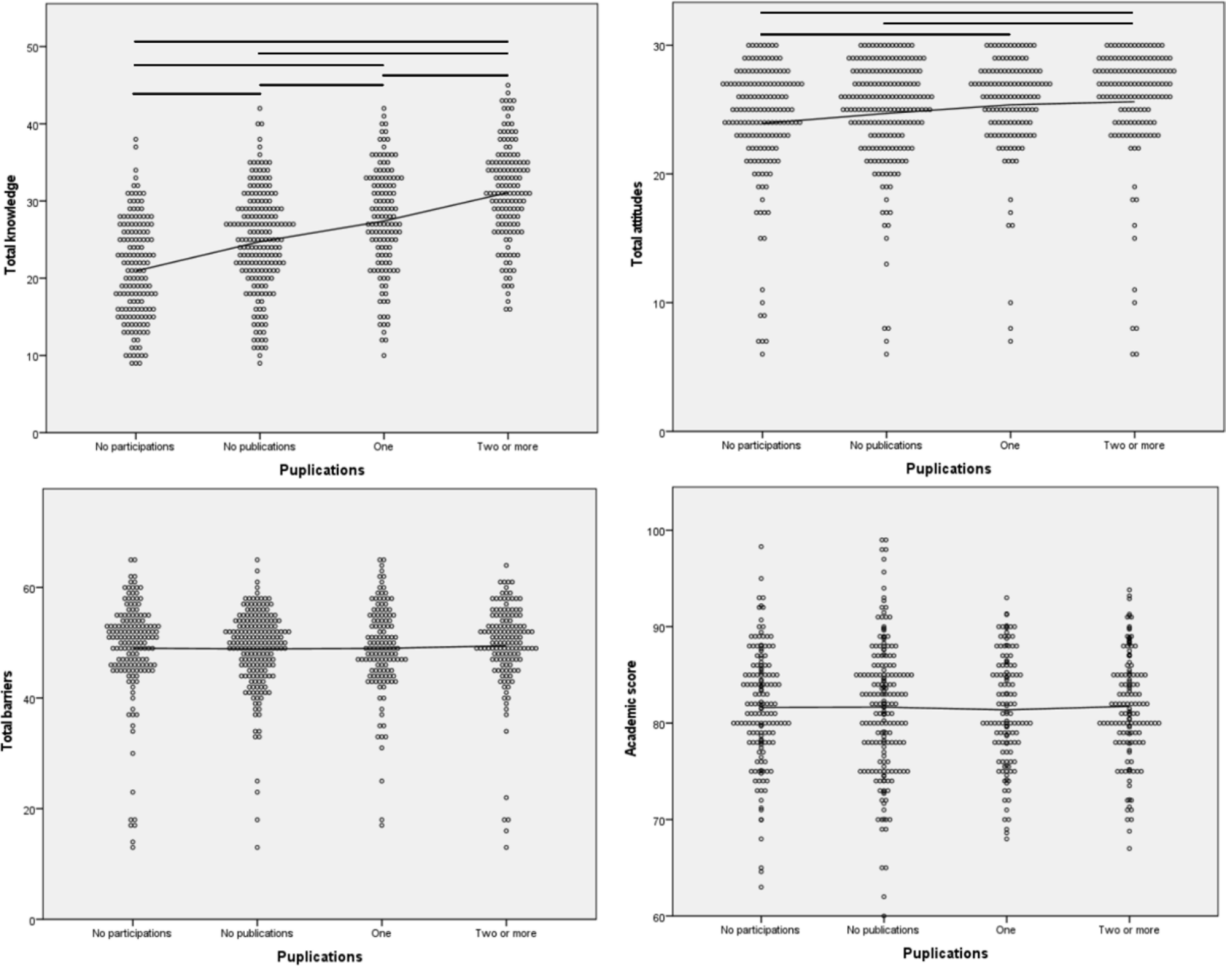
Association between the number of publications and research-related knowledge, attitudes, barriers, and academic scores. Legend: Scatter point plot of participants total knowledge, attitudes, barriers, and academic scores against their published research studies. Independent samples Kruskal-Wallis non-parametric tests were conducted for each graph. An interpolation straight line was plotted for each graph as well. The horizontal lines illustrate only the statistically significant pairwise comparisons when the groups test was statistically significant. The significance level of pairwise comparisons were adjusted for multiple comparisons

The more research projects they were involved in, the higher knowledge scores the participants self-reported (*P* < 0.001). Furthermore, those who participated in more than three research projects had higher attitudes towards research than all the other arms (*P* < 0.001 for all). In contrast, perceived barriers and academic scores were not significantly different between participants who were involved in different numbers of research studies (Fig. 1). Knowledge and attitude scores for published authors were significantly higher than those without publications (*P* < 0.001 for both), in comparison to barriers and academic scores, which were similar among all groups (Fig. 2). Further analysis, specifically focusing on participants who successfully finalized and submitted a minimum of three research articles (*n* = 273), revealed that individuals reporting eventual acceptance of most or all their submitted articles for publication exhibited higher knowledge and attitudes scores compared to their counterparts who reported otherwise (*P* < 0.001 for both; Supplementary Fig. 1). Our sample also showed moderate correlation between knowledge and attitudes (rho = 0.39; *P* < 0.001; Supplementary Fig. 2), which also showed moderate (rho = 0.48) and weak (0.20) correlations with English competence, respectively (*P* < 0.001 for both; Supplementary Fig. 3). Conversely, academic scores did not correlate with any of the knowledge, attitudes, and barriers towards medical research (Supplementary Fig. 4).

Ordinal logistic regression analysis of a model that consisted of knowledge, attitudes, and barriers items could explain 46% and 34% of the variability in research involvement and publications, respectively (*P* < 0.001). Nevertheless, a model of knowledge alone could also explain 41% of the variance in research involvement and 29% for publications (*P* < 0.001). Knowledge about the publication process (Regression coefficient = 0.69; its 95%CI [0.50–0.89]), planning and conducting research (0.49 [0.25–0.73]), searching the medical literature (0.44 [0.21–0.67]), and academic writing (0.26 [0.04–0.48]) were significant independent predictors of research involvement. On the other hand, the independent predictors for publications were knowing the publication process (0.79 [0.56–1.02]) and identifying topics with lack of evidence (0.31 [0.04–0.59]). Surprisingly, knowledge of statistical analysis was a significant negative independent predictor for both involvement (-0.29 [-0.48, -0.10]) and publication (-0.35 [-0.58, -0.12]) of research studies. Moreover, the perception that “research is not always costly” was the most important independent attitude predictor for participation and publication (0.26 [0.12–0.40]) and (0.21 [0.03–0.38]), respectively. Regarding barriers, lack of opportunities (-0.17 [0.33, -0.01]) and (-0.30 [0.49, -0.12]) in addition to language and internet limitations (-0.18 [0.32, -0.05]) and (-0.18 [0.34, -0.02]) were independent predictors that negatively affected involvement in and publication of research, respectively (Table 3).

**Fig. 3:**
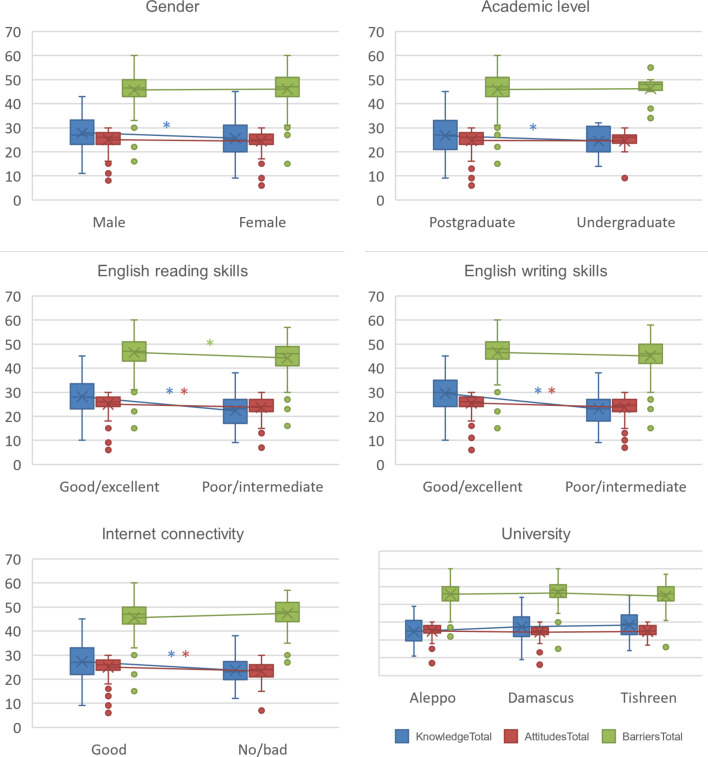
The relationship between demographic characteristics and the total scores of research-related knowledge, attitudes, and barriers. Legend: The * refers to a statistically significant difference in the variable with the matching color; each box depicts an interquartile range; the horizontal line in the box presents the median; the X sign shows the mean; the extending vertical line illustrates the range; the dots that exceed the line represent outliers

As a secondary aim of this study, we evaluated the association between these factors and the demographic characteristics, and we found that male and postgraduate participants had in average a higher research-related knowledge (*P* < 0.01 for both). Superior English competence and internet connectivity were associated with higher knowledge and attitudes towards research (*P* < 0.01 for all, Fig. 3). To study the impact of mentors’ support and sufficient training on the attitudes and barriers towards research, we provided further analysis using data from a previous publication [16], which showed that postgraduates who reported receiving support by their mentors or additional training (mostly extracurricular and self-paced), showed better attitudes towards, and reported less barriers around, research (Supplementary Fig. 5).

## Discussion

This study is the first of its kind to compare self-assessed knowledge, attitudes, and barriers towards research among the Syrian medical students and fresh graduates grouped according to their level of involvement in medical research. First, we found that the larger the number of research projects our participants conducted or published, the higher their self-reported levels of knowledge and attitudes were. The results also indicate that demographic characteristics, such as the male gender and higher self-reported English competence, are linked to greater knowledge and positive attitudes toward research.

Research-related knowledge and attitudes are positively intercorrelated, and correlate with the number of conducted and published research projects. This aligns with previous studies showing that enhancing attitudes towards research augments knowledge and increases research contributions [26, 27]. However, we previously showed that attitudes towards research in Syrian under- and post-graduates are high even in those who were never involved in research activities [16, 18, 28]. This may be an indication for the need for a curated curriculum aimed at improving the foundational research-related knowledge needed to equip students with the knowledge and skills to be productively involved in research. This conclusion is also supported by our findings for the group that participated in two or three research studies; they possessed a greater level of knowledge than their peers with fewer contributions while their attitudes were not higher. Another explanation for the disparity in the relationship between knowledge and attitudes toward research productivity could be that participants’ attitudes, unlike their knowledge, may also have associations with various factors, including language and training level, as well as time barriers in conducting research, especially in the context of insufficient supervision [19, 29–31]. Indeed, these limitations can result in attitudes plateauing while knowledge may continue to advance with increased research contributions.

Conversely, the summed perceived barriers did not show a significant association with research involvement and publication. This could be due to the various unrelated barriers students might face with the different research studies they contribute to. For instance, clinical researchers conducting clinical trials in our resource-limited environment struggle to handle patients’ attitudes towards research, and their adherence to therapies [32]. On the other hand, those conducting retrospective analyses face more issues with suboptimal medical records and documentation [19]. Also, students of healthcare-related domains (e.g., pharmacy or dentistry) are less likely to face a lack of mentors in comparison to medical students due to the different professor-to-student ratios in their respective schools. Similarly, academic scores did not differ among the study arms, which contradicts previous studies that showed more publications by students with higher academic scores [33, 34]. One potential explanation might be the lack of sufficient research training in the official curricula of the Syrian universities and the reliance on extracurricular training to fulfill this gap [19, 28, 31].

A robust understanding of medical research, as well as staying current in a field, are likely important for research productivity. Accordingly, we found a strong correlation between the number of publications and the level of experience in the publication process and in identifying gaps in the literature and topics for research. Experience in these necessary practical skills was still rated as below average for a large proportion of our sample, identifying a clear need for educational interventions. This may also partially explain the low acceptance rates for research articles submitted from Syria [18]. However, not all practical skillsare directly linked to lapses in productivity. For instance, the finding of the negative correlation between self-reported statistical analysis skills and the publication rate may reflect greater awareness of authors about their weakness [35]. Interestingly, the mere total score of knowledge items studied in this report explained a great portion of the variability in research conduction and publication.Given that the studied knowledge items are practical skills that can be relatively easy to modify, an opportunity for improvement here is a clear starting point. For instance, prior interventions have demonstrated an enhancement in understanding research methodology [36, 37], academic writing [28, 31, 38], publication processes [31], and searching the medical literature [36].

The most important perceived barriers towards contributing to and publishing research studies were the lack of opportunities and training as well as language barriers. The former is a common obstacle of research in the region [29, 39], which is unsurprising in settings with limited research supervision [18], where students and residents need to self-train and create their own opportunities [19]. On the other hand, English competency, which correlated with higher knowledge and attitudes towards research in this study, is likely important in several ways, not limited to being the dominant language for self-paced education [16]. Good English command is also crucial for developing good scientific writing skills, having better understanding and appraisal of the literature [24], and participating in international collaborations [40, 41].

Several demographic factors associated with higher research-related knowledge including gender and internet connectivity. Internet accessibility is a basic infrastructural need for consuming, producing, and training in medical research [19, 28, 31, 36, 42]. However, basic internet accessibility is not a demanding asset to be supplied across the medical facilities in Syria especially with the accumulating evidence about the effectiveness of asynchronous virtual training on research related skills [31, 37]. On the other hand, gender inequities in medicine have been widely identified in literature with slower academic progress for female faculty members compared to men [43]. Therefore, more attention should be paid to gender equality in this challenging field of medicine, especially as the attitudes towards research in our sample were comparable between females and males in contrast to the higher attitudes found among males in other studies [44, 45].

Finally, we showed a significant association between the favorable attitudes towards research and mentors’ support as well as self-paced extracurricular training. Worldwide, mentors’ support remains an essential factor in the research productivity of their students [16, 23, 30] and their tendency to pursue academic careers [26, 46]. Although this support is challenging in the Syrian example with the scarcity of available mentors [16, 18], it can still be achieved by collaborations with mentors overseas or by targeting the already available mentors with interventionsaimed at increasing their own attitudes and capacities in supporting the research projects of their mentees [1, 47]. On the other hand, allowing more space for extracurricular medical-research initiatives among early career healthcare professionals can have a huge impact on their practical skills and research motivation [8, 19, 36, 48–51]. They may also strengthen the independence among early career researchers and reduce their reliance on university’s mentors [1].

This study was not free of limitations. As with any observational and survey study, all reported relationships between research productivity and the knowledge, attitudes, and barriers scores were associations that cannot confer any causality. Additionally, these knowledge, attitudes, and barriers were self-assessed and reported, reflecting the participants’ opinions rather than being objective measures. Furthermore, the survey administration relied on an online approach, which may have led to potential selection bias. Finally, the questionnaire used in this study has only undergone face validation. This limitation is due to the absence of validated tools in the field and the lack of resources necessary to conduct more comprehensive validation processes. Nevertheless, this study is unique due to its distinctive approach in selectively sampling published authors, encompassing individuals across diverse academic levels and healthcare specialties across Syria.

## Conclusion

Research productivity of medical students and residents in Syria correlated with their practical research-related knowledge and positive attitudes towards research. Although academic performance and the perceived barriers towards research did not correlate with research contributions nor with participants’ knowledge or attitudes, self-reported superior English competency correlated with all of them. Additionally, the male gender and stable internet connectivity were associated with higher participants’ knowledge and motivation towards research. Finally, these findings may indicate a potential opportunity for improvement by focusing on enhancing extracurricular training interventions and mentors’ support.

### Electronic supplementary material

Below is the link to the electronic supplementary material.

Supplementary Material 1

## Data Availability

The complete dataset is available and can be supplemented upon request to the corresponding author.

## References

[1] El Achi N, Papamichail A, Rizk A, Lindsay H, Menassa M, Abdul-Khalek RA (2019). A conceptual framework for capacity strengthening of health research in conflict: the case of the Middle East and North Africa region. Global Health.

[2] Lansang MA, Dennis R (2004). Building capacity in health research in the developing world. Bull World Health Organ.

[3] Sumathipala A, Siribaddana S, Patel V (2004). Under-representation of developing countries in the research literature: ethical issues arising from a survey of five leading medical journals. BMC Med Ethics.

[4] Alhaffar BA, Abbas G, Alhaffar AA (2019). The prevalence of burnout syndrome among resident physicians in Syria. J Occup Med Toxicol.

[5] Ashkar K, Romani M, Musharrafieh U, Chaaya M (2010). Prevalence of burnout syndrome among medical residents: experience of a developing country. Postgrad Med J.

[6] Pokhrel NB, Khadayat R, Tulachan P (2020). Depression, anxiety, and burnout among medical students and residents of a medical school in Nepal: a cross-sectional study. BMC Psychiatry.

[7] Ratnakaran B, Prabhakaran A, Karunakaran V (2016). Prevalence of burnout and its correlates among residents in a tertiary medical center in Kerala, India: a cross-sectional study. J Postgrad Med.

[8] Assar A, Matar SG, Hasabo EA, Elsayed SM, Zaazouee MS, Hamdallah A (2022). Knowledge, attitudes, practices and perceived barriers towards research in undergraduate medical students of six arab countries. BMC Med Educ.

[9] Naicker S, Plange-Rhule J, Tutt R, Eastwood J (2009). Shortage of Healthcare Workers in developing Countries—Africa. Ethn Dis.

[10] Diab MM, Taftaf RMO, Arabi M (2011). Research productivity in Syria: quantitative and qualitative analysis of current status. Avicenna J Med.

[11] Matar H, Almerie Q, Adams C, Essali A (2009).

[12] Sahloul MZ, Monla-Hassan J, Sankari A, Kherallah M, Atassi B, Badr S (2016). War is the enemy of Health. Pulmonary, critical care, and Sleep Medicine in War-Torn Syria. Https://DoiOrg/101513/AnnalsATS201510-661PS.

[13] Stone-Brown K. Syria: a healthcare system on the brink of collapse. BMJ. 2013;347. 10.1136/BMJ.F7375. 10.1136/bmj.f7375 24327182

[14] Coutts A, Fouad FM (2013). Response to Syria’s health crisis—poor and uncoordinated. Lancet.

[15] Alahdab F, Omar MH, Alsakka S, Al-Moujahed A, Atassi B (2014). Syrians’ alternative to a health care system: field hospitals. Avicenna J Med.

[16] Hanafi I, Haj Kassem L, Hanafi M, Ahmad S, Abbas O, Hajeer MY, et al. Medical Research Conduct and Publication during Higher Education in Syria: attitudes, barriers, practices, and possible solutions. Avicenna J Med. 2022;12. 10.1055/S-0042-1755387. 10.1055/s-0042-1755387 PMC9458349 36092380

[17] Alahdab F, Firwana B, Hasan R, Sonbol MB, Fares M, Alnahhas I (2012). Undergraduate medical students’ perceptions, attitudes, and competencies in evidence-based medicine (EBM), and their understanding of EBM reality in Syria. BMC Res Notes.

[18] Turk T, Al Saadi T, Alkhatib M, Hanafi I, Alahdab F, Firwana B (2018). Attitudes, barriers, and practices toward research and publication among medical students at the University of Damascus, Syria. Avicenna J Med.

[19] Al Saadi T, Abbas F, Turk T, Alkhatib M, Hanafi I, Alahdab F (2018). Medical research in war-torn Syria: medical students’ perspective. Lancet.

[20] Amin TT, Kaliyadan F, Al Qattan EA, Al Majed MH, Al Khanjaf HS, Mirza M. Knowledge, attitudes and barriers related to participation of medical students in research in three arab universities. Educ Med J. 2012;4. 10.5959/EIMJ.V4I1.7.

[21] Albahari D, Bashir M (2020). Gender gap in mental health research productivity: results from Qatar. Asian J Psychiatr.

[22] El-Ouahi J, Larivière V (2023). On the lack of women researchers in the Middle East and North Africa. Scientometrics.

[23] Mitwalli HA, Al Ghamdi KM, Moussa NA (2014). Perceptions, attitudes, and practices towards research among resident physicians in training in Saudi Arabia. East Mediterr Health J.

[24] Wieczorek ALigia Mitręga, Maciej. CeDeWu. Academic teachers under stress in the Publish or Perish Era. Int J Oper Prod Manage 2017.

[25] Association WM (2013). World Medical Association Declaration of Helsinki: ethical principles for Medical Research Involving human subjects. JAMA.

[26] Zier K, Stagnaro-Green A. A multifaceted program to encourage medical students’ research. Acad Med 2001;76. 10.1097/00001888-200107000-00021 11448834

[27] Houlden RL, Raja JB, Collier CP, Clark AF, Waugh JM (2004). Medical students’ perceptions of an undergraduate research elective. Med Teach.

[28] Sabouni A, Chaar A, Bdaiwi Y, Masrani A, Abolaban H, Alahdab F (2017). An online academic writing and publishing skills course: help syrians find their voice. Avicenna J Med.

[29] AlGhamdi KM, Moussa NA, AlEssa DS, AlOthimeen N, Al-Saud AS (2014). Perceptions, attitudes and practices toward research among senior medical students. Saudi Pharm J.

[30] Memarpour M, Fard AP, Ghasemi R. Evaluation of attitude to, knowledge of and barriers toward research among medical science students. Asia Pac Fam Med. 2015;14. 10.1186/S12930-015-0019-2. 10.1186/s12930-015-0019-2 PMC4336721 25705121

[31] Hanafi I, Kheder K, Sabouni R, Gorra Al Nafouri M, Hanafi B, Alsalkini M, et al. Improvingacademic writing in a low-resource country: a systematic examination of online peer-run training. Teach LearnMed. 2024;1–15. 10.1080/10401334.2024.2332890. 10.1080/10401334.2024.2332890 38551184

[32] Djurisic S, Rath A, Gaber S, Garattini S, Bertele V, Ngwabyt SN et al. Barriers to the conduct of randomised clinical trials within all disease areas. Trials 2017;18. 10.1186/S13063-017-2099-9. 10.1186/s13063-017-2099-9 PMC5539637 28764809

[33] Seaburg LA, Wang AT, West CP, Reed DA, Halvorsen AJ, Engstler G (2016). Associations between resident physicians’ publications and clinical performance during residency training. BMC Med Educ.

[34] Schott NJ, Emerick TD, Metro DG, Sakai T (2013). The cost of resident scholarly activity and its effect on resident clinical experience. Anesth Analg.

[35] Hayat MJ, Schwartz TA, Kim M, Ali SZ, Jiroutek MR (2021). A comparative cross-sectional assessment of statistical knowledge of faculty across five health science disciplines. J Clin Transl Sci.

[36] Alahdab F, Alabed S, Al-Moujahed A, Al Sallakh MA, Alyousef T, Alsharif U (2017). Evidence-based medicine: a persisting desire under fire. BMJ Evid Based Med.

[37] Sabouni A, Bdaiwi Y, Janoudi SL, Namous LO, Turk T, Alkhatib M (2017). Multiple strategy peer-taught evidence-based medicine course in a poor resource setting. BMC Med Educ.

[38] Gaber SA, Ali SI (2022). Effectiveness of a training program in improving scientific writing skills based on APA 7 style among Postgraduate Students. Int J Learn Teach Educational Res.

[39] Abdel-Kareem A, Kabbash I, Saied S, Al-Deeb A (2019). Knowledge, practices and attitudes of physicians towards evidencebased medicine in Egypt. East Mediterr Health J.

[40] Curry MJ, Lillis TM (2010). Academic research networks: accessing resources for English-medium publishing. Engl Specif Purp.

[41] Abouzeid M, Elzalabany MK, Nuwayhid I, Jabbour S. Conflict-related health research in Syria, 2011–2019: a scoping review for the Lancet - AUB Commission on Syria. Confl Health 2021;15. 10.1186/S13031-021-00384-3. 10.1186/s13031-021-00384-3 PMC8672497 34906178

[42] Habineza H, Nsanzabaganwa C, Nyirimanzi N, Umuhoza C, Cartledge K, Conard C, et al. Perceived attitudes of the importance and barriers to research amongst Rwandan interns and pediatric residents – a cross-sectional study. BMC Med Educ. 2019;19. 10.1186/S12909-018-1425-6. 10.1186/s12909-018-1425-6 PMC6318911 30606184

[43] Chitsamatanga B, Rembe S, Shumba J, Chitsamatanga B (2019). Barriers to Research and Publication efforts of female academics: a case of selected universities in South Africa and Zimbabwe. Anthropologist.

[44] Bilal M, Haseeb A, Mari A, Ahmed S, Sher Khan MA, Saad M (2019). Knowledge, attitudes, and barriers toward Research among Medical students of Karachi. Cureus.

[45] Khan H, Khan S, Iqbal A (2009). Knowledge, attitudes and practices around health research: the perspective of physicians-in-training in Pakistan. BMC Med Educ.

[46] DeLong MR, Hughes DB, Tandon VJ, Choi BD, Zenn MR (2014). Factors influencing fellowship selection, career trajectory, and academic productivity among plastic surgeons. Plast Reconstr Surg.

[47] McGrail MR, Rickard CM, Jones R (2006). Publish or perish: a systematic review of interventions to increase academic publication rates. Http://DxDoiOrg/101080/07294360500453053.

[48] Burgoyne LN, O’Flynn S, Boylan GB (2010). Undergraduate medical research: the student perspective. Med Educ Online.

[49] Brimo Alsaman MZ, Sallah H, Badawi R, Ghawi A, Shashaa MN, Kassem LH (2021). Syrian medical, dental and pharmaceutical publication in the last decade: a bibliometric analysis. Ann Med Surg (Lond).

[50] Busse CE, Anderson EW, Endale T, Smith YR, Kaniecki M, Shannon C (2022). Strengthening research capacity: a systematic review of manuscript writing and publishing interventions for researchers in low-income and middle-income countries. BMJ Glob Health.

[51] Hanafi I, Alsalkini M, Kheder K, Al Nafouri MG, Rahmeh AR, Sabouni R. Analyzing theefficacy of a decade-long endeavor: extracurricular medical research training amidst the turmoil of Syria. medRxiv. 2024. 10.1101/2024.03.17.24304430.

