# Supplementary material for: Factors influencing research productivity among Syrian medical professionals amidst conflict: a case-control study
Source: BMC Med Educ. 2024 Jul 11;24:747. doi: 10.1186/s12909-024-05681-y (PMC11241956; doi:10.1186/s12909-024-05681-y)
Supplement: Supplementary file 1 — Supplementary Material 1 [file 12909_2024_5681_MOESM1_ESM.docx]

Supplementary Figure 1: The association between the rate of papers acceptance for publication and participants’ knowledge, attitudes, barriers towards research, and academic scores.


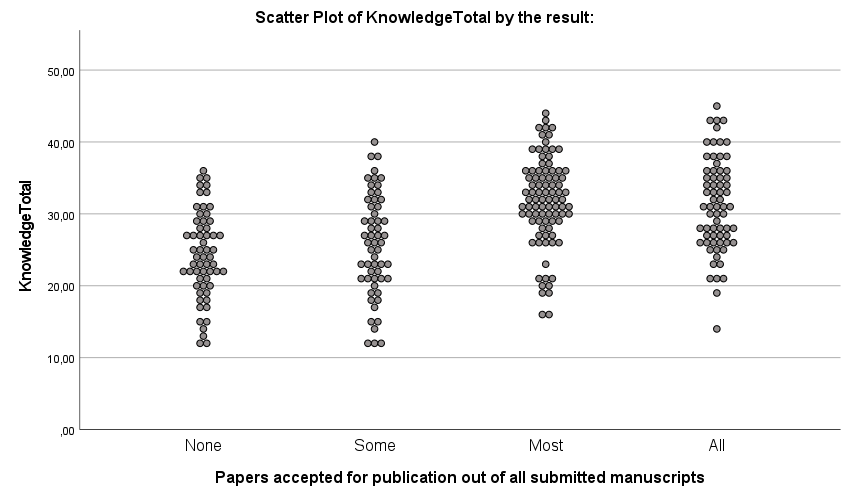

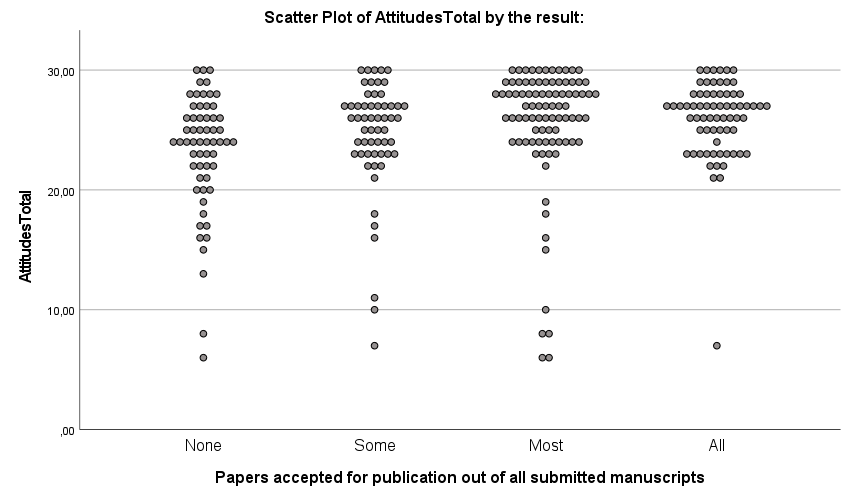

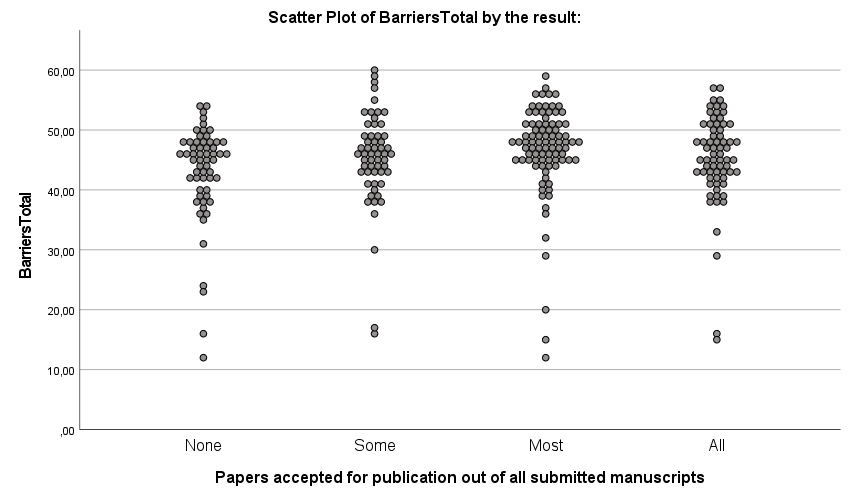

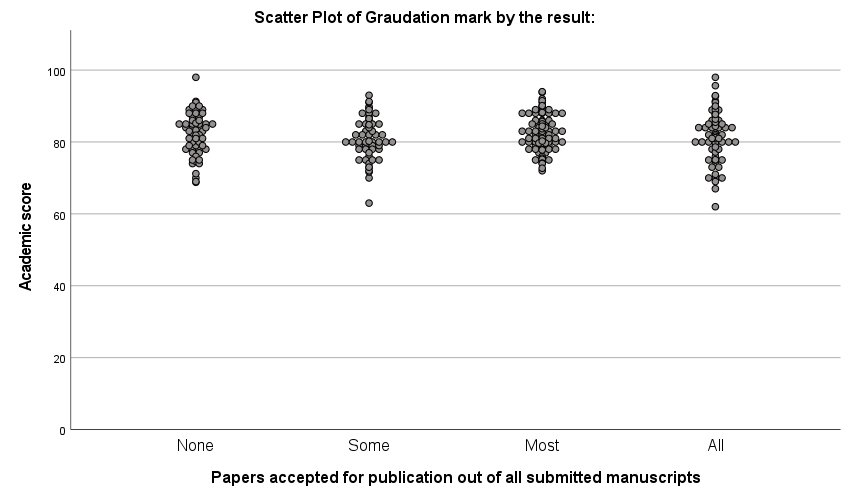


Supplementary Figure 1 legend: Scatter point plot of participants total knowledge, attitudes, barriers, and academic scores against the rate of acceptance of their submitted manuscripts. Independent samples Kruskal-Wallis non-parametric tests were conducted for each graph. The horizontal lines illustrate only the statistically significant pairwise comparisons when the groups’ test was statistically significant. The significance level of pairwise comparisons were adjusted for multiple comparisons.

Supplementary Figure 2: Correlations among participants’ knowledge, attitudes, and barriers towards medical research.


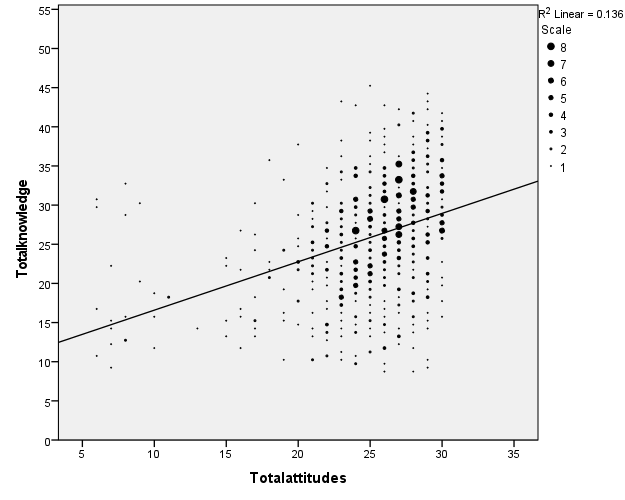

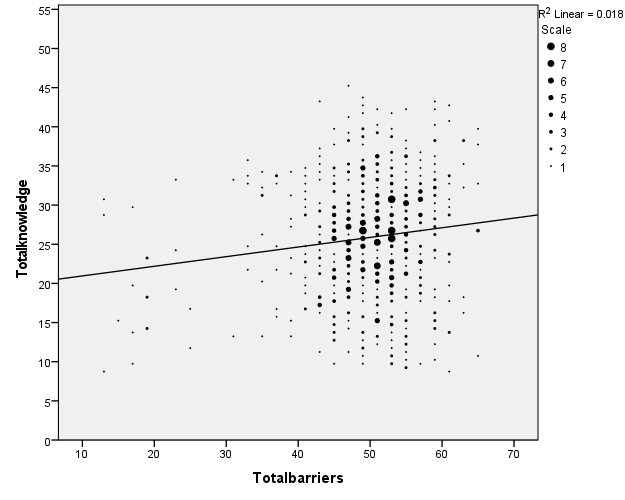

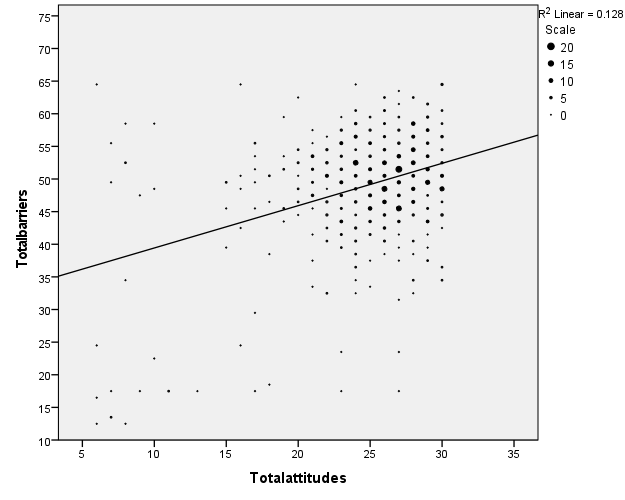


| Spearman’s rho correlation | | P value |
| --- | --- | --- |
| Knowledge Vs. attitudes | 0.39 | <0.001 |
| Knowledge Vs. barriers | 0.10 | 0.019 |
| Barriers Vs. attitudes | 0.16 | <0.001 |

Supplementary Figure 2 Legend: Correlation between the total scores of knowledge, attitudes, and barriers of the whole sample. All three correlations were statistically significant with the values summarized in the attached table.

Supplementary Figure 3: Correlations between participants’ English language skills and their academic scores, knowledge, attitudes, and barriers towards medical research.

| Spearman’s rho correlation | | P value |
| --- | --- | --- |
| Knowledge | 0.48 | <0.001 |
| Attitudes | 0.20 | <0.001 |
| Barriers | 0.10 | 0.010 |
| Academic scores | -0.12 | 0.004 |


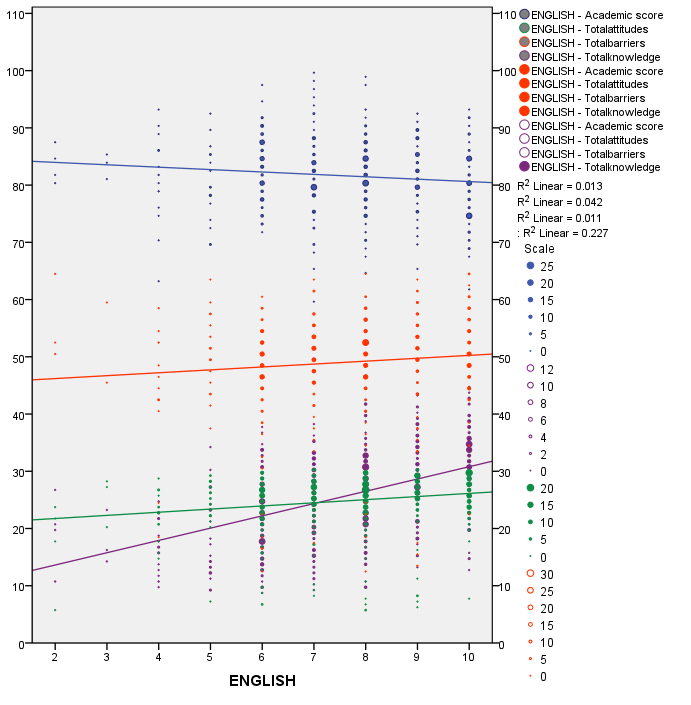


Supplementary Figure 3 Legend: Correlations between English language skills and the total scores of knowledge, attitudes, barriers, and academic scores of the whole sample. All four correlations were statistically significant with the values summarized in the attached table. The X-axis represent the sum of the two five-point Likert scores for self-reported English reading and writing skills.

Supplementary Figure 4: Correlations between participants’ academic scores and their knowledge, attitudes, and barriers towards medical research.


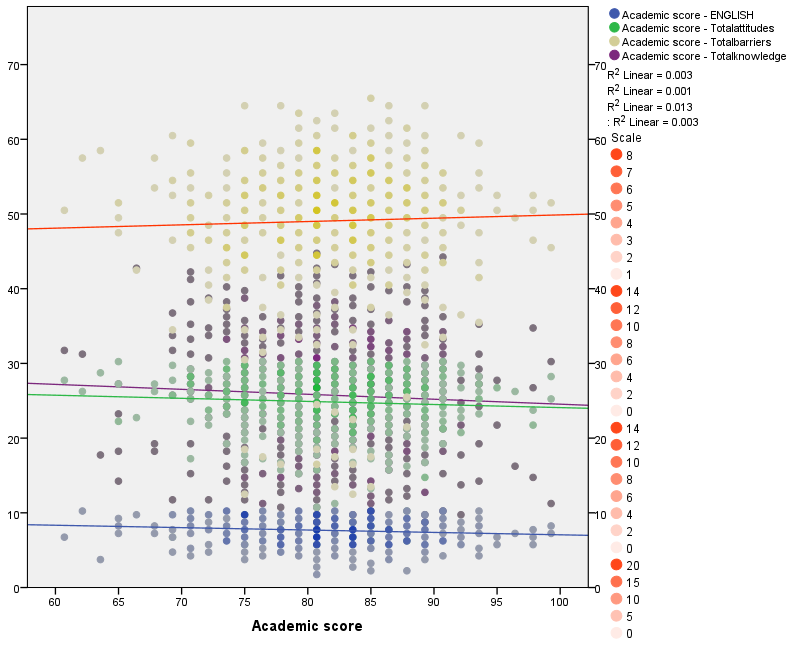


| Spearman’s rho correlation | | P value |
| --- | --- | --- |
| Knowledge | -0.05 | 0.187 |
| Attitudes | -0.06 | 0.166 |
| Barriers | 0.03 | 0.412 |
| English skills | -0.12 | 0.004 |

Supplementary Figure 4 Legend: Correlations between academic scoers and the total scores of knowledge, attitudes, barriers, as well as English total skills (i.e., as calculated in supplementary figure 2) of the whole sample.

Supplementary Figure 5: A supplementary analysis on the data of a previous publication (Hanafi et al. 2022) assessing the impact of sufficient research training and mentors’ encouragement on the attitudes and perceived barriers towards medical research among postgraduates.


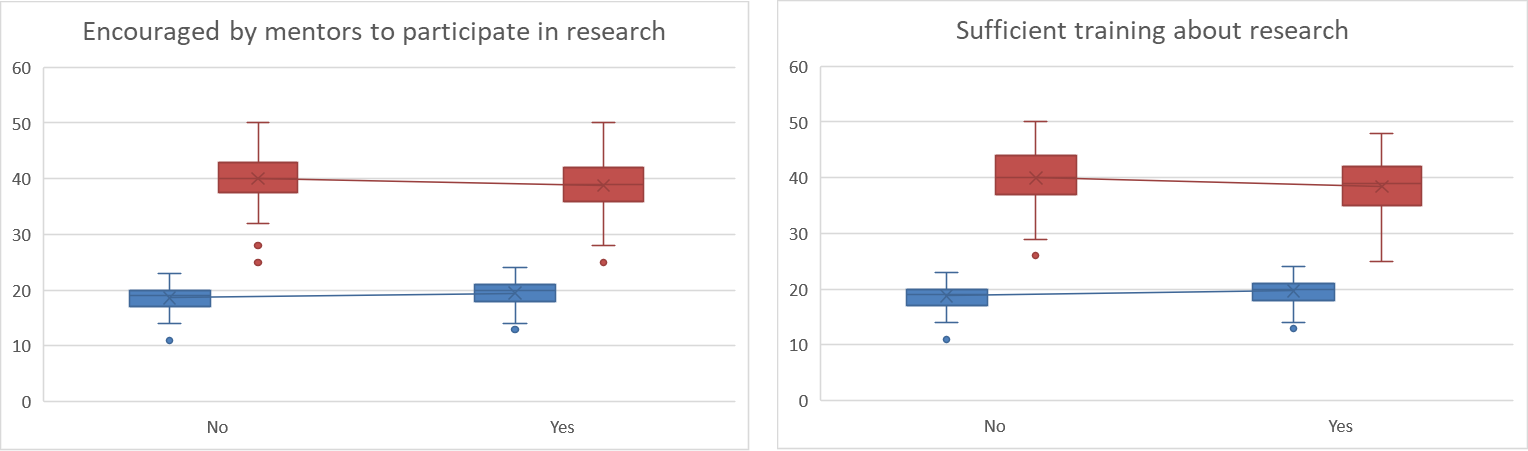

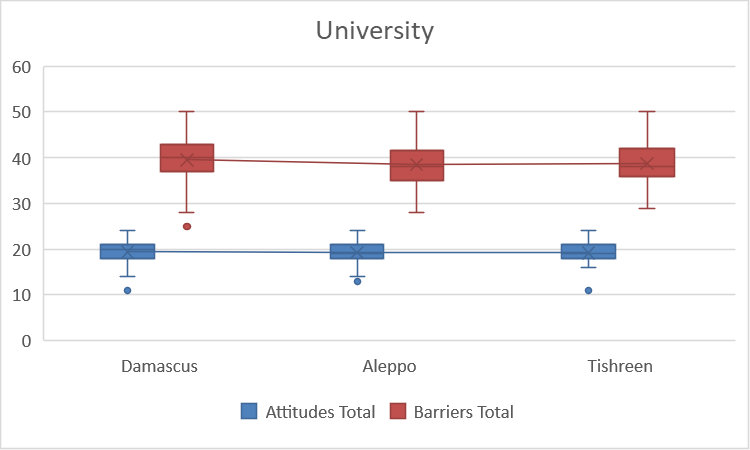


*

*

*

Supplementary Figure 5 legend: The * refers to a statistically significant difference in the variable with the matching color; each box depicts an interquartile range; the horizontal line in the box presents the median; the X sign shows the mean; the extending vertical line illustrates the range; the dots that exceed the line represent outliers.
